# Ethyl 5,8-dibromo-2-dibromo­methyl-6,7-dimeth­oxyquinoline-3-carb­oxy­late

**DOI:** 10.1107/S1600536810029351

**Published:** 2010-07-31

**Authors:** Ting Zhou, Yu-hua Long, Ding-qiao Yang, Han-mei Zhang, Wen-ling Wang

**Affiliations:** a School of Chemistry and Environment, South China Normal University, Guangzhou 510006, People’s Republic of China

## Abstract

The title compound, C_15_H_13_Br_4_NO_4_, was obtained *via* radical bromination reaction of ethyl 6,7-dimeth­oxy-2-methyl­quinoline-3-carboxyl­ate and *N*-bromo­succinimide (NBS) in the presence of benzoyl peroxide (BPO) under photocatalytic conditions. The quinoline ring system is approximately planar with a maximum deviation from the mean plane of 0.035 (1) Å. The dihedral angle between the six-membered rings is 2.33 (2)°. The meth­oxy O atoms of the two neighboring meth­oxy groups are in-plane while their methyl C atoms are located on either side of the quinolyl ring plane at distances of −1.207 (1) and 1.223 (1) Å.

## Related literature

The quinoline nucleus is widely present in numerous natural compounds, see: Michael *et al.* (1997 ▶, 2002 ▶). For the biological activity of quinoline derivatives, see: Heath *et al.* (2004 ▶); Keyaerts *et al.* (2004 ▶); Ko *et al.* (2001 ▶). For our previous work on the preparation of quinoline derivatives, see: Yang *et al.* (2007 ▶, 2008 ▶).
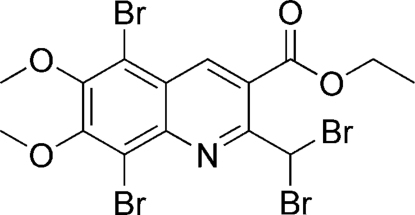


## Experimental

### 

#### Crystal data

C_15_H_13_Br_4_NO_4_
*M* *_r_* = 590.90
Triclinic, 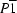

*a* = 8.992 (2) Å
*b* = 9.632 (2) Å
*c* = 11.454 (3) Å
α = 84.868 (3)°
β = 71.948 (3)°
γ = 77.552 (3)°
*V* = 920.8 (4) Å^3^
*Z* = 2
Mo *K*α radiation
μ = 8.76 mm^−1^
*T* = 298 K
0.25 × 0.20 × 0.18 mm

#### Data collection

Bruker APEXII CCD area detector diffractometer
Absorption correction: multi-scan (*SADABS*; Bruker, 2002 ▶) *T*_min_ = 0.218, *T*_max_ = 0.302
4750 measured reflections
3257 independent reflections
2373 reflections with *I* > 2σ(*I*)
*R*_int_ = 0.035

#### Refinement

*R*[*F* ^2^ > 2σ(*F* ^2^)] = 0.043
*wR*(*F* ^2^) = 0.105
*S* = 0.97
3257 reflections
220 parameters
H-atom parameters constrained
Δρ_max_ = 0.97 e Å^−3^
Δρ_min_ = −0.74 e Å^−3^

### 

Data collection: *APEX2* (Bruker, 2004 ▶); cell refinement: *SAINT* (Bruker, 2004 ▶); data reduction: *SAINT*; program(s) used to solve structure: *SHELXS97* (Sheldrick, 2008 ▶); program(s) used to refine structure: *SHELXL97* (Sheldrick, 2008 ▶); molecular graphics: *SHELXTL* (Sheldrick, 2008 ▶); software used to prepare material for publication: *SHELXL97* and *PLATON* (Spek, 2009 ▶).

## Supplementary Material

Crystal structure: contains datablocks quinoline, I. DOI: 10.1107/S1600536810029351/si2277sup1.cif

Structure factors: contains datablocks I. DOI: 10.1107/S1600536810029351/si2277Isup2.hkl

Additional supplementary materials:  crystallographic information; 3D view; checkCIF report

## Figures and Tables

**Fig. 1.**
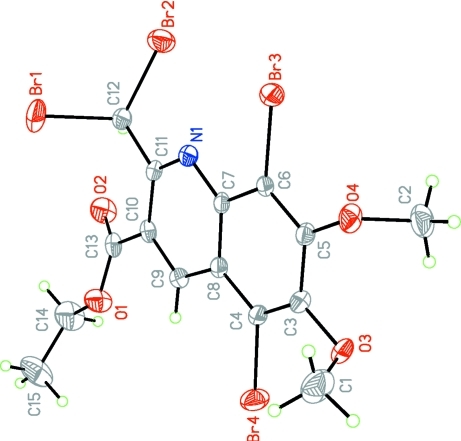
Molecular structure of the title compound showing the atom-labelling scheme. Displacement ellipsoids are drawn at the 50% probability level.

**Fig. 2.**
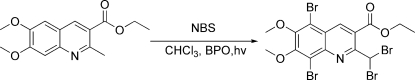
Synthesis of the title compound.

## supplementary crystallographic information

### Comment

The quinoline ring system is widely present in numerous natural compounds (Michael *et al.*, 1997, 2002). Quinoline derivatives are pharmacologically active compounds displaying a wide range of biological activity (Heath *et al.*, 2004; Keyaerts *et al.*, 2004; Ko *et al.*, 2001)).

In previous works, we have reported the synthesis of some new quinoline derivatives (Yang *et al.*, 2007, 2008). Herein, we report the synthesis and structure determination of a new Bromine-containing quinoline derivative, resulting from the radical bromination of ethyl 6,7-dimethoxy-2-methylquinoline-3-carboxylate under photocatalytic conditions. Our attempt to brominate the methyl group linked at C-2 position of quinoline ring, which has an acetal function at C-3, failed and led to 5,8-Dibromo-2-dibromomethyl-6,7- dimethoxy-quinoline-3-carboxylic acid, ethyl ester and other by-pruducts. This compound is the result of a unwanted reaction.

The molecular geometry of the title compound is illustrated in Fig 1. The title molecule contains an approximate planar quinolyl moiety with a maximum deviation from the mean plane of 0.035 (1)Å. The dihedral angle between the six-membered rings is 2.33 (2)°. The methoxy O atoms of the two neighboring methoxy groups are in-plane and their methyl C atoms locate on both sides of the quinolyl ring plane with maximun out-of-plane deviations of -1.207 (1) and 1.223 (1)Å, respectively.

### Experimental

The title compoud was syntheized by treating 1mmol of ethyl 6,7-dimethoxy-2-methylquinoline-3-carboxylate with 1.5mmol of *N*-bromosuccinimide (NBS) in presence of 0.5mmol of Benzoyl Peroxide (BPO) in CCl3 under photocatalytic conditions. The mixture was then cooled and filtered off and the filtrate was concentrated under reduced pressure. The crude product was purified by silica gel column chromatography with the gradient mixture of petroleum ether and ethyl acetate (v : v = 30 : 1) to afford the white product. Crystals suitable for X-ray analysis were obtained by slow evaporation of the mixted solution of petroleum ether and ethyl acetate of the title compound.

### Refinement

The H atoms were positioned geometrically and allowed to ride on their parent atoms, with C—H = 0.93–0.98 Å, with *U*_iso_(H) = 1.2 *U*_eq_(C) or 1.5*U*_eq_(C_methyl_).

### Crystal data

**Table:**

C_15_H_13_Br_4_NO_4_	*Z* = 2
*M**_r_* = 590.90	*F*(000) = 564
Triclinic, *P*1	*D*_x_ = 2.131 Mg m^−^^3^
Hall symbol: -P 1	Mo *K*α radiation, λ = 0.71073 Å
*a* = 8.992 (2) Å	Cell parameters from 3257 reflections
*b* = 9.632 (2) Å	θ = 1.9–25.2°
*c* = 11.454 (3) Å	µ = 8.76 mm^−^^1^
α = 84.868 (3)°	*T* = 298 K
β = 71.948 (3)°	Block, colourless
γ = 77.552 (3)°	0.25 × 0.20 × 0.18 mm
*V* = 920.8 (4) Å^3^	

### Data collection

**Table:**

Bruker APEXII CCD area detector diffractometer	3257 independent reflections
Radiation source: fine-focus sealed tube	2373 reflections with *I* > 2σ(*I*)
graphite	*R*_int_ = 0.035
phi and ω scans	θ_max_ = 25.2°, θ_min_ = 1.9°
Absorption correction: multi-scan (*SADABS*; Bruker, 2002)	*h* = −10→10
*T*_min_ = 0.218, *T*_max_ = 0.302	*k* = −8→11
4750 measured reflections	*l* = −13→13

### Refinement

**Table:**

Refinement on *F*^2^	Primary atom site location: structure-invariant direct methods
Least-squares matrix: full	Secondary atom site location: difference Fourier map
*R*[*F*^2^ > 2σ(*F*^2^)] = 0.043	Hydrogen site location: inferred from neighbouring sites
*wR*(*F*^2^) = 0.105	H-atom parameters constrained
*S* = 0.97	*w* = 1/[σ^2^(*F*_o_^2^) + (0.0511*P*)^2^] where *P* = (*F*_o_^2^ + 2*F*_c_^2^)/3
3257 reflections	(Δ/σ)_max_ = 0.001
220 parameters	Δρ_max_ = 0.97 e Å^−^^3^
0 restraints	Δρ_min_ = −0.74 e Å^−^^3^

### Special details

**Table:**

Geometry. All esds (except the esd in the dihedral angle between two l.s. planes) are estimated using the full covariance matrix. The cell esds are taken into account individually in the estimation of esds in distances, angles and torsion angles; correlations between esds in cell parameters are only used when they are defined by crystal symmetry. An approximate (isotropic) treatment of cell esds is used for estimating esds involving l.s. planes.
Refinement. Refinement of F^2^ against ALL reflections. The weighted R-factor wR and goodness of fit S are based on F^2^, conventional R-factors R are based on F, with F set to zero for negative F^2^. The threshold expression of F^2^ > 2sigma(F^2^) is used only for calculating R-factors(gt) etc. and is not relevant to the choice of reflections for refinement. R-factors based on F^2^ are statistically about twice as large as those based on F, and R- factors based on ALL data will be even larger.

### Fractional atomic coordinates and isotropic or equivalent isotropic displacement parameters (Å^2^)

**Table:**

	*x*	*y*	*z*	*U*_iso_*/*U*_eq_	
Br1	0.57694 (8)	1.26070 (8)	0.23987 (7)	0.0629 (2)	
Br2	0.89932 (9)	1.37644 (7)	0.12167 (7)	0.0596 (2)	
Br3	1.17101 (7)	1.17543 (6)	0.39139 (6)	0.04485 (19)	
Br4	1.13760 (9)	0.53496 (6)	0.26742 (6)	0.0561 (2)	
C1	1.2668 (10)	0.5760 (8)	0.5327 (7)	0.075 (2)	
H18A	1.2422	0.6536	0.5870	0.112*	
H18B	1.3424	0.4998	0.5547	0.112*	
H18C	1.1710	0.5430	0.5393	0.112*	
C2	1.5111 (8)	0.8569 (7)	0.4025 (6)	0.0580 (18)	
H15A	1.5351	0.7702	0.3592	0.087*	
H15B	1.5688	0.8449	0.4618	0.087*	
H15C	1.5419	0.9323	0.3454	0.087*	
C3	1.2399 (6)	0.7379 (5)	0.3697 (5)	0.0342 (12)	
C4	1.1433 (6)	0.7213 (5)	0.3041 (5)	0.0336 (12)	
C5	1.2496 (6)	0.8762 (5)	0.3958 (5)	0.0314 (12)	
C6	1.1574 (6)	0.9927 (5)	0.3568 (5)	0.0298 (11)	
C7	1.0550 (6)	0.9772 (5)	0.2876 (4)	0.0283 (11)	
C8	1.0484 (6)	0.8386 (5)	0.2603 (5)	0.0304 (12)	
C9	0.9471 (6)	0.8295 (5)	0.1901 (5)	0.0345 (12)	
H19	0.9363	0.7405	0.1723	0.041*	
C10	0.8643 (6)	0.9490 (5)	0.1478 (5)	0.0333 (12)	
C11	0.8822 (6)	1.0830 (5)	0.1777 (5)	0.0321 (12)	
C12	0.7951 (6)	1.2193 (5)	0.1337 (5)	0.0364 (13)	
H14	0.7917	1.2035	0.0513	0.044*	
C13	0.7654 (7)	0.9337 (6)	0.0670 (5)	0.0397 (13)	
C14	0.6348 (9)	0.7820 (7)	0.0037 (6)	0.072 (2)	
H16A	0.7074	0.7516	−0.0760	0.086*	
H16B	0.5601	0.8673	−0.0079	0.086*	
C15	0.5464 (10)	0.6653 (8)	0.0669 (7)	0.082 (3)	
H17A	0.6213	0.5837	0.0820	0.123*	
H17B	0.4910	0.6396	0.0152	0.123*	
H17C	0.4709	0.6988	0.1435	0.123*	
N1	0.9716 (5)	1.0961 (4)	0.2468 (4)	0.0330 (10)	
O1	0.7234 (5)	0.8094 (4)	0.0843 (4)	0.0535 (12)	
O2	0.7315 (5)	1.0223 (5)	−0.0062 (4)	0.0556 (12)	
O3	1.3345 (5)	0.6235 (4)	0.4075 (4)	0.0436 (10)	
O4	1.3441 (5)	0.8917 (4)	0.4638 (3)	0.0429 (10)	

### Atomic displacement parameters (Å^2^)

**Table:**

	*U*^11^	*U*^22^	*U*^33^	*U*^12^	*U*^13^	*U*^23^
Br1	0.0304 (4)	0.0793 (5)	0.0675 (5)	0.0064 (3)	−0.0113 (3)	0.0034 (4)
Br2	0.0622 (5)	0.0435 (4)	0.0804 (5)	−0.0165 (3)	−0.0315 (4)	0.0111 (3)
Br3	0.0409 (4)	0.0368 (3)	0.0633 (4)	−0.0074 (3)	−0.0231 (3)	−0.0080 (3)
Br4	0.0643 (5)	0.0319 (3)	0.0826 (5)	−0.0068 (3)	−0.0370 (4)	−0.0071 (3)
C1	0.069 (6)	0.070 (5)	0.076 (6)	0.002 (4)	−0.027 (5)	0.025 (4)
C2	0.038 (4)	0.073 (5)	0.072 (5)	−0.013 (3)	−0.023 (4)	−0.016 (3)
C3	0.030 (3)	0.031 (3)	0.038 (3)	0.000 (2)	−0.009 (3)	−0.003 (2)
C4	0.027 (3)	0.028 (3)	0.048 (3)	−0.003 (2)	−0.013 (3)	−0.005 (2)
C5	0.025 (3)	0.033 (3)	0.036 (3)	−0.002 (2)	−0.011 (2)	−0.003 (2)
C6	0.020 (3)	0.029 (3)	0.040 (3)	−0.005 (2)	−0.007 (2)	−0.006 (2)
C7	0.018 (3)	0.033 (3)	0.032 (3)	−0.003 (2)	−0.006 (2)	−0.003 (2)
C8	0.023 (3)	0.032 (3)	0.036 (3)	−0.005 (2)	−0.008 (2)	−0.003 (2)
C9	0.030 (3)	0.033 (3)	0.042 (3)	−0.007 (2)	−0.012 (3)	−0.001 (2)
C10	0.022 (3)	0.044 (3)	0.037 (3)	−0.009 (2)	−0.010 (2)	−0.002 (2)
C11	0.021 (3)	0.032 (3)	0.039 (3)	0.000 (2)	−0.006 (2)	−0.002 (2)
C12	0.030 (3)	0.037 (3)	0.043 (3)	−0.004 (2)	−0.015 (3)	0.001 (2)
C13	0.023 (3)	0.053 (4)	0.043 (3)	−0.006 (3)	−0.010 (3)	−0.005 (3)
C14	0.079 (6)	0.084 (5)	0.083 (5)	−0.030 (5)	−0.057 (5)	−0.003 (4)
C15	0.089 (7)	0.104 (6)	0.091 (6)	−0.060 (5)	−0.060 (5)	0.021 (5)
N1	0.028 (3)	0.033 (2)	0.038 (3)	−0.004 (2)	−0.013 (2)	0.0012 (19)
O1	0.064 (3)	0.051 (2)	0.068 (3)	−0.021 (2)	−0.045 (3)	0.004 (2)
O2	0.060 (3)	0.063 (3)	0.061 (3)	−0.017 (2)	−0.042 (3)	0.008 (2)
O3	0.033 (2)	0.036 (2)	0.060 (3)	0.0060 (18)	−0.021 (2)	0.0009 (18)
O4	0.037 (3)	0.051 (2)	0.049 (2)	−0.0027 (19)	−0.026 (2)	−0.0097 (18)

### Geometric parameters (Å, °)

**Table:**

Br1—C12	1.939 (6)	C7—N1	1.358 (6)
Br2—C12	1.919 (5)	C7—C8	1.415 (6)
Br3—C6	1.876 (4)	C8—C9	1.409 (6)
Br4—C4	1.894 (5)	C9—C10	1.367 (7)
C1—O3	1.449 (8)	C9—H19	0.9300
C1—H18A	0.9600	C10—C11	1.418 (7)
C1—H18B	0.9600	C10—C13	1.504 (7)
C1—H18C	0.9600	C11—N1	1.322 (6)
C2—O4	1.426 (7)	C11—C12	1.507 (7)
C2—H15A	0.9600	C12—H14	0.9800
C2—H15B	0.9600	C13—O2	1.200 (6)
C2—H15C	0.9600	C13—O1	1.313 (6)
C3—C4	1.353 (7)	C14—O1	1.465 (6)
C3—O3	1.365 (6)	C14—C15	1.517 (11)
C3—C5	1.417 (7)	C14—H16A	0.9700
C4—C8	1.415 (7)	C14—H16B	0.9700
C5—O4	1.355 (5)	C15—H17A	0.9600
C5—C6	1.373 (7)	C15—H17B	0.9600
C6—C7	1.425 (6)	C15—H17C	0.9600
			
O3—C1—H18A	109.5	C8—C9—H19	119.4
O3—C1—H18B	109.5	C9—C10—C11	118.0 (4)
H18A—C1—H18B	109.5	C9—C10—C13	119.2 (5)
O3—C1—H18C	109.5	C11—C10—C13	122.8 (5)
H18A—C1—H18C	109.5	N1—C11—C10	122.7 (5)
H18B—C1—H18C	109.5	N1—C11—C12	116.4 (4)
O4—C2—H15A	109.5	C10—C11—C12	120.9 (4)
O4—C2—H15B	109.5	C11—C12—Br2	113.1 (3)
H15A—C2—H15B	109.5	C11—C12—Br1	109.2 (4)
O4—C2—H15C	109.5	Br2—C12—Br1	111.5 (3)
H15A—C2—H15C	109.5	C11—C12—H14	107.6
H15B—C2—H15C	109.5	Br2—C12—H14	107.6
C4—C3—O3	121.2 (4)	Br1—C12—H14	107.6
C4—C3—C5	120.0 (5)	O2—C13—O1	123.9 (5)
O3—C3—C5	118.7 (4)	O2—C13—C10	124.7 (5)
C3—C4—C8	122.2 (4)	O1—C13—C10	111.4 (5)
C3—C4—Br4	118.8 (4)	O1—C14—C15	106.3 (4)
C8—C4—Br4	119.0 (3)	O1—C14—H16A	110.5
O4—C5—C6	120.8 (4)	C15—C14—H16A	110.5
O4—C5—C3	119.6 (4)	O1—C14—H16B	110.5
C6—C5—C3	119.6 (4)	C15—C14—H16B	110.5
C5—C6—C7	121.1 (4)	H16A—C14—H16B	108.7
C5—C6—Br3	119.4 (3)	C14—C15—H17A	109.5
C7—C6—Br3	119.5 (4)	C14—C15—H17B	109.5
N1—C7—C8	122.5 (4)	H17A—C15—H17B	109.5
N1—C7—C6	118.7 (4)	C14—C15—H17C	109.5
C8—C7—C6	118.7 (4)	H17A—C15—H17C	109.5
C9—C8—C4	125.3 (4)	H17B—C15—H17C	109.5
C9—C8—C7	116.4 (4)	C11—N1—C7	119.2 (4)
C4—C8—C7	118.3 (4)	C13—O1—C14	115.0 (4)
C10—C9—C8	121.2 (5)	C3—O3—C1	114.0 (5)
C10—C9—H19	119.4	C5—O4—C2	114.8 (4)
			
O3—C3—C4—C8	−177.7 (5)	C8—C9—C10—C11	−0.6 (8)
C5—C3—C4—C8	−0.5 (8)	C8—C9—C10—C13	176.4 (5)
O3—C3—C4—Br4	1.2 (7)	C9—C10—C11—N1	−1.7 (8)
C5—C3—C4—Br4	178.4 (4)	C13—C10—C11—N1	−178.7 (5)
C4—C3—C5—O4	178.6 (5)	C9—C10—C11—C12	179.8 (5)
O3—C3—C5—O4	−4.1 (7)	C13—C10—C11—C12	2.8 (8)
C4—C3—C5—C6	1.6 (8)	N1—C11—C12—Br2	26.6 (6)
O3—C3—C5—C6	178.9 (5)	C10—C11—C12—Br2	−154.9 (4)
O4—C5—C6—C7	−178.5 (5)	N1—C11—C12—Br1	−98.2 (5)
C3—C5—C6—C7	−1.6 (8)	C10—C11—C12—Br1	80.3 (5)
O4—C5—C6—Br3	3.2 (7)	C9—C10—C13—O2	−154.5 (6)
C3—C5—C6—Br3	−179.9 (4)	C11—C10—C13—O2	22.4 (9)
C5—C6—C7—N1	−177.2 (5)	C9—C10—C13—O1	24.0 (7)
Br3—C6—C7—N1	1.1 (7)	C11—C10—C13—O1	−159.1 (5)
C5—C6—C7—C8	0.5 (7)	C10—C11—N1—C7	2.5 (8)
Br3—C6—C7—C8	178.8 (4)	C12—C11—N1—C7	−179.0 (5)
C3—C4—C8—C9	179.0 (5)	C8—C7—N1—C11	−0.8 (8)
Br4—C4—C8—C9	0.1 (7)	C6—C7—N1—C11	176.8 (5)
C3—C4—C8—C7	−0.6 (8)	O2—C13—O1—C14	2.1 (9)
Br4—C4—C8—C7	−179.5 (4)	C10—C13—O1—C14	−176.4 (5)
N1—C7—C8—C9	−1.4 (7)	C15—C14—O1—C13	−159.2 (6)
C6—C7—C8—C9	−179.0 (5)	C4—C3—O3—C1	−97.1 (6)
N1—C7—C8—C4	178.2 (5)	C5—C3—O3—C1	85.7 (6)
C6—C7—C8—C4	0.5 (7)	C6—C5—O4—C2	−111.0 (6)
C4—C8—C9—C10	−177.5 (5)	C3—C5—O4—C2	72.0 (6)
C7—C8—C9—C10	2.1 (8)		
